# Unleashing the mysterious link between COVID-19 and a famous childhood vasculitis: Kawasaki disease

**DOI:** 10.1186/s43054-020-00029-9

**Published:** 2020-07-01

**Authors:** Antoine Fakhry AbdelMassih, Aisha Said AbdelAzeam, Aya Ayad, Aya Yasser Kamel, Ayah Khalil, Basma Kotb, Dina Waheed, Esraa Menshawey, Fady Sefein, Farah Taha, Habiba-Allah Ismail, Ibrahim Osman, John Iskander, Lama El Wakil, Lara Rashad, Mariem Badr Arsanyous, Meryam El Shershaby, Mina Mansour, Mirette Ashraf, Nada Hafez, Nadeen Mohamed Abuzeid, Noheir Mahmoud-Nashaat AbdElSalam, Nouran Gamal Hafez, Nourhan Youssef, Rafeef Hozaien, Rana Saeed, Dina Kamel, Manal Ahmed AbdelHameed, Salma Ali

**Affiliations:** 1 Pediatrics’ Department, Pediatric Cardiology Unit, Cairo University Children Hospital, Faculty of Medicine, Cairo University, Kasr Al Ainy street, Cairo, 12411 Egypt; 2 Students’ and Interns’ Research Program (Research accesibility team), Faculty of Medicine, Cairo University, Al-Saray Street, El Manial, PO BOX: 11956, Cairo, Egypt; 3 Resident training Program, Faculty of Medicine, Cairo University, Al-Saray Street, El Manial, PO BOX: 11956, Cairo, Egypt; 4 Pediatrics’ Department, Faculty of Medicine, Ain Shams University, Abbassyia, PO BOX: 11566, Cairo, Egypt; 5 Pediatrics’ Department, Faculty of Medicine, Cairo University, Al-Saray Street, El Manial, PO BOX: 11956, Cairo, Egypt

**Keywords:** COVID-19, Kawasaki disease, Shared genetic and inflammatory pathways, Upregulated inflammation

## Abstract

**Background:**

Coronavirus disease 2019 (COVID-19) emerged as a small outbreak in Wuhan rapidly progressing into the deadliest pandemic since the Spanish flu of 1918. The disease was deemed trivial in children, until the reporting, few days ago, of an emerging pediatric multi-inflammatory syndrome mimicking Kawasaki disease (KD).

**Main body:**

This report reveals that coronaviridae were implicated in induction of several post-infectious vasculitides, namely, KD, AHEI, and HSP. This occurs in genetically susceptible individuals to vascular inflammation. Shared genetic susceptibilities between KD and CoV include genes encoding for CD 40, HLAB-15:03, and ACE. This leads to augmented inflammation with hypersecretion of cytokines especially IL-6.

**Conclusion:**

The revealed relationships between KD and CoV can help to predict the risk of KD in COVID-19 patients through screening levels of upregulated cytokines. It might also signify that classic treatment of KD with IVIG might need to be replaced with anti-cytokine therapy in COVID-19 patients.

## Background

Coronavirus disease 2019 (COVID-19) is caused by the seventh member of the Coronaviridea (CoV) family, severe acute respiratory syndrome coronavirus-2 (SARS-CoV-2). It was declared a public health emergency of international concern on January 30, 2020, and was identified as an outbreak in March 2020. Millions of cases have been reported worldwide, with no fully effective vaccine or treatment up to this moment. It has not been widely identified in pediatrics, and the aggregated data on children with COVID-19 are rare. Ludvigsson et al. [1] in a systematic review stated that about 2% of the 44,672 confirmed cases of COVID-19 were children aged 0–19 years, of which 0.9% were under 10 years of age at diagnosis. They also stated that children often have a milder form of the disease than adults with better prognosis and fewer death rates. However, New York health officials began issuing alerts on May 4, describing cases of young patients, between the ages of 2 and 15, who presented with inflammation in multiple organ systems, and features of Kawasaki disease (KD), a childhood illness of unclear origin. On May 6, the number of suspected cases increased to 64, and within that week, three children had died. Concurrently, France’s health minister Olivier Véran told parliament that there had been some 15 cases of KD in France, although not all of the cases tested positive for COVID-19. National Health Service (NHS) of England recently warned of a similar syndrome being increasingly common in their hospital admissions [2, 3]. To date, only one case report has been published displaying features of KD and testing positive for COVID-19. The 6-month-old patient case reported by Jones et al. [4] presented with fever, refusal to eat, and minimal or no respiratory symptoms as cough, rhinorrhea, and congestion. The subsequent day, she developed an erythematous not pruritic, blotchy rash, and 2 days later, her findings escalated to tachycardia, tachypnea, a fever of 38.3 °C, limb-sparing conjunctivitis, and dry cracked lips. On the fifth day of her fever, she was diagnosed with KD and treated accordingly with intravenous immunoglobulins (IVIG) and high dose aspirin. Prior to admission, she tested positive for COVID-19 and was discharged 2 days later being advised to quarantine at home for 14 days while taking low-dose aspirin provided that her echocardiogram revealed no abnormalities.

It is poorly understood if both diseases occurred concurrently, or if there is rather a causal relationship between both illnesses. KD is an acute febrile illness of unknown cause that primarily affects children, especially boys younger than 5 years of age. It is one of the known causes of fever of unknown origin (FUO) in children. The disease was first described in Japan by Tomisaku Kawasaki in 1967. Typical diagnostic criteria include fever, rash, swelling of the hands and feet, peeling of skin, non-purulent conjunctival injection, lymphadenopathy, and coronary artery abnormalities [4, 5].

In this review, we are analyzing previously available data to demonstrate the possible relationship between KD and COVID-19. We hypothesize that such a link might be post-infectious in genetically susceptible patients. We also hypothesize that this relationship operates through an increased tendency for vascular inflammation and upregulated cytokine profile. Finally, yet importantly, we are discussing the possible need for treatment modification of KD developing in the midst of COVID-19.

## Main text

### KD, a possible post-infectious illness, is CoV implicated?

#### Evidence of several respiratory infections inducing KD

According to various studies, the most accepted theory for the etiology of KD is the exposure to one or more infectious agent(s) will trigger an inflammatory response in genetically predisposed individuals [6, 7]. In 2000, Rowley et al. [8] discovered that during acute KD, immunoglobulin A (IgA) plasma cells infiltrate vascular tissues (as coronary walls) and non-vascular tissues, primarily the trachea which highly suggest the route of entry of the KD etiologic agents is respiratory. Also, in another study, Rowley et al. reported oligoclonal IgA antibodies in bronchial epithelium from patients with acute KD in addition to the macrophages found in inflamed coronary artery tissues. This highly supports the idea that the immune response triggered by KD is antigen-driven (hence oligoclonal) [9]. Intracytoplasmic inclusion bodies were also detected indicating a viral etiology for KD [10]. Further investigation of the infectious etiology of KD was done by Chang et al. [11] who found an association between viral infections, such as respiratory viruses, and the development of KD. In addition, Kim et al. [12] stated that a viral infection most likely precedes KD, supporting the theory that KD has a post-infectious etiology. Many studies thereof set out to identify the causative organisms triggering KD, such as Negro et al. [13], citing a higher incidence of developing all six major criteria of KD in patients who had a recent or active parvovirus B19 infection. Weng et al. [14] also noted a significant incidence of KD in children with previously documented enterovirus infection.

The theory that KD is a post-infectious illness has been confirmed by Maggio et al. [15], who found KD and Kawasaki shock syndrome in two siblings after documented parvovirus infections. Schnaar et al. [16] reported similar findings in cousins who were in close contact following parainfluenza type 3 viral infections, and Lee et al. [17] indicated the involvement of varicella-zoster virus in two sisters. Bocavirus was also implicated by Bajolle et al. [18], while the involvement of adenovirus has only been postulated but not proven by Shike et al. [19] due to similar clinical features. The possibility of KD being post-infectious is also proved by Embil et al. [20], who managed to extract adenovirus from the mesenteric lymph nodes in two KD patients post mortem.

#### Coronaviridae epidemiology tracing in KD

There have been studies suggesting the possible involvement of CoV in KD. Serological tests were performed for human coronavirus 229E (HCoV-229E) and supported that a particular strain of the virus might play a role in the etiology of KD [21]. Other studies focused on coronavirus OC43/HKU1 [22] and a case of COVID-19 [23]. While there was also initial speculation of the involvement of COV-NL63, these claims have since been refuted.

Evidence-based immunopathological studies done by Rowley et al. [24] suggest that KD is caused by a respiratory infectious agent. Furthermore, the suspected seasonality of KD roughly coincides with that of the most common respiratory HCoVs [25]. However, it is too early to tell if such seasonality also applies to COVID-19. Further research is necessary, but the factors above suggest a probable connection between CoV and KD.

#### Association of coronaviridae with other types of vasculitides

CoV has a synergistic relationship with patients manifesting post-infectious vasculitis, as vasculitis can be either a risk factor or post-infectious outcome to coronavirus. Owing to the fact that vasculitis can be a consequence to coronavirus, a case report presented by Chesser et al. [26] showed the recurrence of acute hemorrhagic edema of infancy (AHEI) in an 8-month-old female who previously tested positive for coronavirus NL63 via Nucleic Acid Amplification test (NAAT). AHEI is a rare disease affecting infants younger than 24 months, and is known as a benign type of leukocytoclastic vasculitis, and is considered a variant of Henoch Schoenlein Purpura (HSP).

Additionally, from January 2013 till December 2016, the Korean Centers for Disease Control and Prevention gathered information on pathogens causing acute respiratory and enteric infectious diseases, to study the seasonal tendencies in pediatric HSP, and its correlation with the outbreak of infectious diseases. The seasonal analysis indicated that a considerable number of HSP cases (5252, 31.0%, and 4437) were diagnosed in spring and winter respectively, while the smaller number was in summer (3224, 19.0%). This supports that HSP cases are mostly reported in colder months, coinciding with the winter infectious outbreaks as coronavirus [27, 28]. Correlation analysis between Henoch–Schönlein purpura occurrence and prevalence of viral infections in the former study indicated that there were 2184 positive cases of coronavirus, with a *p* value (0.005) and correlation coefficient (0.194). The etiology of HSP remains quite vague; however, upper respiratory tract infections preceded 30% of the HSP cases reported in Spain [27].

A report from China [29] supported the vascular effect of severe acute respiratory syndrome (SARS), by investigating the histopathology from the autopsy of three patients who died from SARS. It showed systemic vasculitis with infiltration of small blood vessels by monocytes, lymphocytes, focal necrosis, and edema in multiple organs including the lung, heart, brain, liver, and kidneys.

### Endothelial inflammation in both disorders

There is an increasing body of evidence that KD involves widespread endothelial dysfunction, such endothelial dysfunction might be induced by reactive oxygen species. It endothelial dysfunction and inflammation in patients with KD is not limited to coronary endothelium but involves renal and mesenteric vascular endothelium and might persist long after resolution of KD [30, 31].

Moreover, there is accumulating evidence showing that the multi-organ failure reported in COVID-19 patients is due mainly to the inflammatory response caused by viral infection of the endothelium rather than to the direct action of the virus. Postmortem examination of vascular samples of affected patients by Varga et al. revealed significant inflammation of the vascular endothelium. This was confirmed by Escher et al., who stated that upregulated cytokines in COVID-19 induce endothelial active inflammation. This might explain the micro-thrombi developing in the lungs of affected patients and the subsequent need of anti-platelets and anticoagulant therapy in most critical patients [32].

### Similar geographic distributions with shared genetics of upregulated inflammation

It is known that KD has higher rates in the far east, namely, in Japan, Korea, Taiwan, and intermediate rates in China, the Philippines, and other Asian countries [33]. Coronavirus outbreaks were first recognized in the Far East, which from there they spread to the whole world. As SARS-CoV started in China, HKU1-CoV started in Hong Kong and Middle East Respiratory Syndrome (MERS)-CoV in the Middle East [34]. Lastly, COVID-19 started in Wuhan, China [35]. The similar geographic and racial distributions of both disorders raise the suspicion of shared genetic susceptibilities in the affected populations.

#### ACE I/D polymorphism

Pooled analysis suggests that the angiotensin converting enzyme insertion/deletion (ACE I/D) polymorphism was significantly associated with KD risk specifically in sub-group analysis by sample size > 200 as demonstrated by Pan et al. [36].

Delanghe et al. mentioned that ACE1 I/D polymorphism may be regarded as a confounder in the spread of COVID-19, and the outcome of the infection in various European populations, where the log-transformed prevalence of COVID-19 infections inversely correlates with the ACE I/D allele frequency. It is worth mentioning that China and Korea, which were initially severely hit by the virus, are characterized by low D allele frequencies [37].

#### Major histocompatibility complex and human leucocyte antigen polymorphism

Major histocompatibility complex (MHC) class I genes and human leukocyte antigen (HLA) A, B, and C individual genetic variation may affect the severity and susceptibility to SARS-CoV-2 as well as KD.

A comprehensive in silico analysis of viral peptide-MHC class I binding affinity across 145 HLA genotypes for all SARS-CoV-2 peptides was conducted by Nguyen et al. showed that HLA-B15:03 has the greatest capacity to present highly conserved SARS-CoV-2 peptides that are shared among common human coronaviruses. This suggests that **HLA-B15:03** could strongly activate T-cell mediated immunity, with subsequent induction of vascular inflammation [38].

As for HLA variability affecting KD, Oh et al. conducted an analysis of the polymorphisms of HLA types. It proved that there was a significant increase in the frequency of **HLA-B15:03**, along with HLA-B35, HLA-B75, and HLA-Cw09 alleles in patients with KD compared with the control healthy group. When the patients with KD were divided into two subgroups, with or without CC, the KD patients without CC showed a significantly increased frequency of HLA-B35, HLA-B75, and HLA-Cw09 alleles as opposed to a decrease in HLA-A26 when compared with the healthy control group. HLA-B15, the shared HLA between SARS and KD, was linked to a higher risk of endothelial inflammation and coronary aneurysms [39].

#### Ligand gene of cluster of differentiation 40

Cluster of differentiation 40 (CD40) is a co-stimulatory protein found on antigen-presenting cells and is required for their activation. The binding of cytotoxic T cells on TH cells to CD40 activates antigen-presenting cells and induces a variety of downstream effects. Such effects have been linked to increased risk of vasculitis and endothelial dysfunction.

Kumrah et al. stated that CD40LG (ligand gene of CD 40) has been found as an independent predictor of KD occurrence and KD progression [40].

SARS-CoV-2 interaction with human cells has been thought to occur exclusively through angiotensin converting enzyme 2 (ACE2). Recently, Dakal et al. stated that SARS-CoV-2 attachment to host cells is possibly mediated also by a sequence of tripeptide consisting of arginine, glycine, and aspartate (RGD). Integrin binding would activate the expression of **CD40LG**; CD40LG augmented expression increases the likelihood of inflammatory sequelae of COVID-19 [41].

### Cytokine profile in both disorders

#### Innate immunity cytokines: IL2, IL10, and TNF

Interlukin-2 (IL-2), interlukin-10 (IL-10), and tumor necrosis factor-alpha (TNF-α) were measured in a study involving intensive care unit (ICU) patients with COVID-19 showed higher significant levels than those who were not admitted [42]. A previously published paper proved that IL-2 receptor level was higher in KD patients than in control group [43].

Moreover, Hsueh et al. proved that IL-10 promotor polymorphism at position 592 (IL-10-592*A allele) located on chromosome 1q31-q32 with KD genetic susceptibility in Taiwanese patients [44]. Also, according to a study on KD pathogenesis, children suffering from the disease presented with significantly elevated levels of TNFα. In addition, anti-TNFα was tested in the treatment of KD and showed significant improvement [45].

#### TGF-β

Simultaneously, the role of transforming growth factor beta (TGF-β) in COVID-19 promotes viral entry into the cells [46]. In the context of Kawasaki, TGF-β affects the genetic susceptibility of KD, regarding the outcome and likelihood for persistent coronary aneurysms. Furthermore, TGF-β modulates the balance of pro-inflammatory/anti-inflammatory T-cells through the expression of the Forkhead box P3 (FOXP3), which affects the differentiation function and survival of CD4+ CD25+ regulatory T cells. Additionally, it causes coronary artery aneurysm by promoting the production of myofibroblast [44].

#### IL-6 a common effector between KD and COVID-19

Initiated by tumor necrotic factor alpha (TNF-α), IL-6, a pro-inflammatory cytokine, and an anti-inflammatory myokine are immediately stimulated and secreted in a state of infection or tissue injury, which greatly contributes to the hosts’ defense mechanisms [47, 48]. This is via activation of the IL-6 gene, which will result in the arousal of acute-phase responses and immune reactions as proven by experiments done in vivo and vitro [47].

The contribution of IL-6 with regards to KD has not been clearly understood. It is presumed that IL-6 directly promotes maturation of megakaryocytes, thus responsible for the thrombocytosis witnessed in the disease. Elevated IL-6 inhibits T-helper 1 (Th1) cell differentiation and promotes T-helper 2 (Th2) cell activation, which increases Th2 cytokines; as a result, polyclonal B cell producing autoantibodies will be stimulated. Not only does it contribute to the acute inflammatory reaction of KD, but it also plays a role in its vasculitis component by activation of antibody-mediated endothelial damage [49, 50].

According to several studies, there are increased levels of serum IL-6 in children with KD, especially during the acute phase in comparison to the subacute phase. KD patients with higher levels of IL-6 are more prone to developing coronary artery lesions (CALs), which goes in accordance with Alolika et al. who states that during pre- and post-IVIG treatment, IL-6 levels were drastically greater in KD patients with CALs (143.60 ± 99.72 pg/dL) than those without (52.90 ± 36.46 pg/dL). IL-6 levels were also predictive of the occurrence of coronary aneurisms, with a sensitivity of 83.3% and a specificity of 95.8% [51].

Wu and colleagues mention a unique correlation between increased IL-6 levels and IVIG resistance, suggesting that IL-6 could be a reliable prognostic marker for IVIG resistance. There is a clear difference in the levels of IL-6 in patients without IVIG resistance (64.1 ± 51.5 pg/dL) than those with resistance(184 ± 65.0 pg/dL) [52]. In 2017, a study revealed that elevated IL-6 is an independent risk factor of IVIG non-responsiveness with a sensitivity of 76.19% and a specificity of 61.59%. This is in accordance with Nandi et al., which advocate that IL-6 levels should be used to determine the method of treatment in KD patients. The IVIG mechanism of inflammatory modulation may be largely dependent on Th2 cytokines including IL-6. Whether or not IL-6 is a key factor in the pathogenesis of KD is not crystal clear and requires extensive research to fully understand. As for what is understood, serum IL-6 has proved to be a novel marker for the prediction of coronary artery involvement and resistance to IVIG [53].

In COVID-19, three pathways of inflammations have been identified, namely, the ACE2 and JAK/STAT and Notch pathways. Both pathways have been linked to an increase in IL-6. Evidence suggests that IL-6 levels have been linked to most of the multi-system complications of COVID-19. An elevated response of IL-6-induced severe respiratory distress. Thus, our results suggest that serial measurement of circulating IL-6 levels may be important in identifying disease progression among COVID-19-infected patients. AbdelMassih et al. suggested that IL-6 might be a possible inducer of COVID-19 myocardial complications. Therefore, it is reasonable to suggest that immediate initial evaluation of IL-6 level be performed upon hospital admission of COVID-19 pediatric patients, due to its potential benefits to assess worsening clinical features, and disease progression in COVID-19 [54–61].

### Anti-IL6 therapy and its possible role in COVID-19 induced KD

The repurposed use of anti-IL-6 inhibitors, whether siltuximab, sarlizumab, or tocilizumab, has been identified as a potential therapy for COVID-19 critical patients. Such inhibitors have been linked to decreased oxygen needs and decreased number of mechanical ventilation days compared to symptomatic treatment. This has led to Food and Drug Administration (FDA) approval of several clinical trials involving the use of those drugs for the treatment of critically ill COVID-19 patients [62].

In KD, cytokine-blockade therapies have been suggested as superior therapies to IVIG. A clinical trial of anakinra, IL-1β–blockade therapy, was recently launched (ClinicalTrials.gov number, NCT02390596). IL-6 plays an important role in tissue regeneration, and the targeted blockade of IL-6 could disrupt this process. In addition, IL-6 contributes to the reduction of neutrophil trafficking into the arterial wall. Tocilizumab can inhibit vascular endothelial growth factor, which enhances proliferation and migration of endothelial cells, and contributes to the remodeling of coronary-artery aneurysms [63]. Thus, the use of tocilizumab in the context of COVID-19 patients might be needed, in IVIG resistant cases or even prior to the administration of IVIG.

**Fig. 1:**
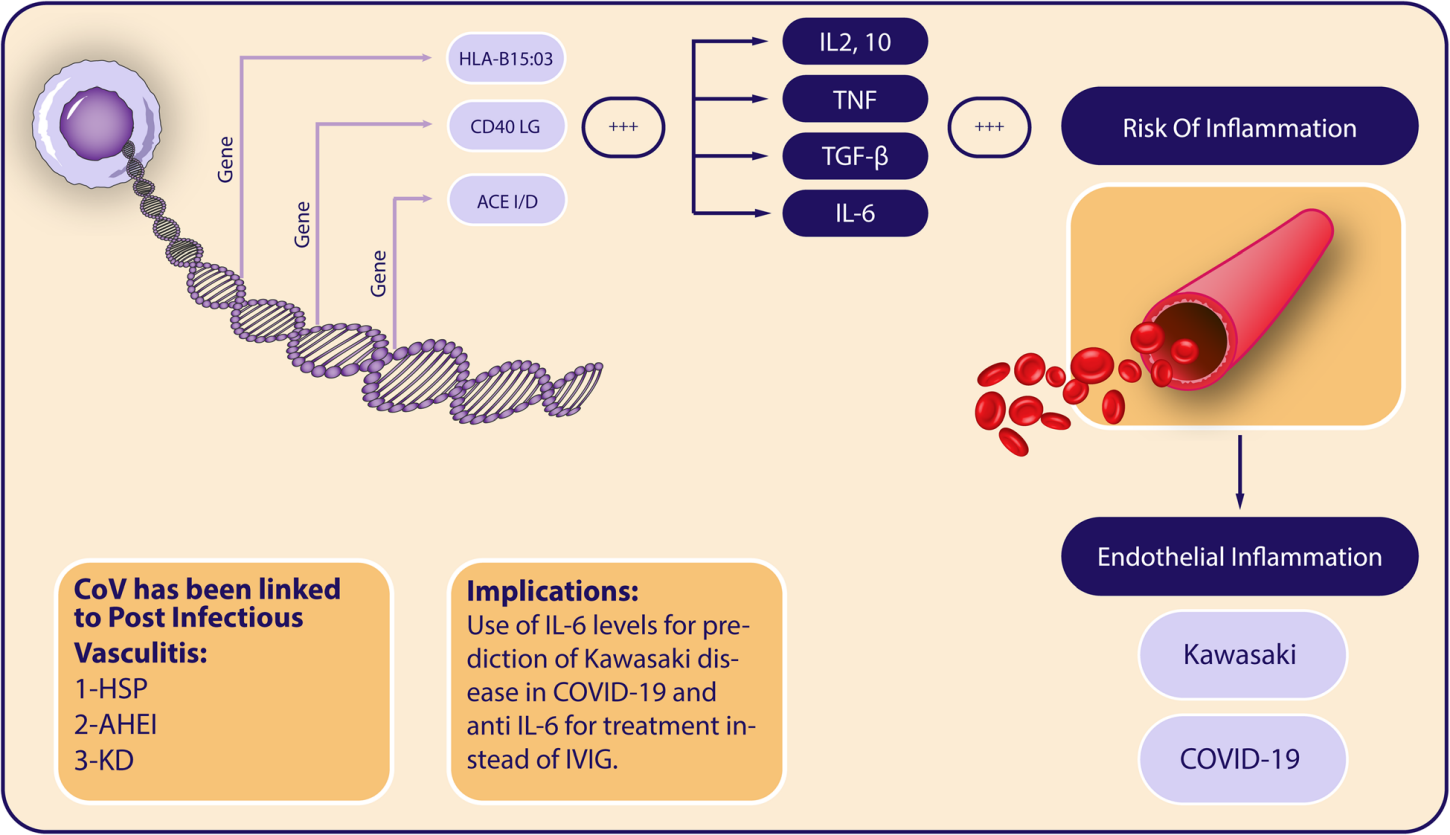
Summary of possible linkage pathways between Kawasaki disease and COVID-19. HLA, human leukocyte antigen; CD4, cluster of differentiation 4; ACE, angiotensin-converting enzyme; IL, interleukin; TNF, tumor necrosis factor; TGF-beta, transforming growth factor beta; HSP, Henoch-Schonlein purpura; AHEI, acute hemorrhagic edema of infancy; KD, Kawasaki disease; IVIG, intravenous immunoglobulin

Figure 1 is a summary of the possible shared mechanisms of KD and COVID-19 and the implications of such findings.

## Conclusion

This is by far, the earliest report to study the linkage between the commonest childhood vasculitis, KD, and COVID-19. There is strong past and present body of evidence that CoV can induce a state of post-infectious vasculitis in genetically predisposed individuals. This occurs through upregulated baseline levels of inflammation. The knowledge of that can help in tailoring the therapeutic strategies in patients presenting with KD concurrently with COVID-19. The early screening of IL-6 levels in pediatric COVID-19 patients can be of utmost importance in detection of subsequent risk of KD. The use of IL-6 inhibitors in those developing KD can have a superior effect that the classic IVIG therapy.

Prospective studies are needed to confirm or refute such findings.

## Data Availability

Not applicable

## Abbreviations

Aspirin: Acetylsalicylic acid
AHEI: Acute hemorrhagic edema of infancy
ACE: Angiotensin converting enzyme
ACE I/D: Angiotensin converting enzyme insertion/deletion
CALs: Coronary artery lesions
CC: Coronary complications
CD: Cluster of differentiation
CD40LG: Ligand gene of CD 40
CoV: Coronaviridae
CRP: C-reactive protein
ESR: Erythrocyte sedimentation rate
FDA: Food and Drug Administration
FOXP3: Forkhead/winged-helix transcription factor P3
FUO: Fever of unknown origin
HCoV: Human coronaviridae
HLA: Human leucocyte antigen
HSP: Henoch-Schönlein purpura
ICU: Intensive care unit
Ig: Immunoglobulin
IL: Interleukin
IVIG: Intravenous immunoglobulins
JAK: Janus kinase
KD: Kawasaki disease
LG: Ligand
MHC: Major histocompatibility complex
NAAT: Nucleic acid amplification test
RGD: Arginine, glycine, and aspartate tripeptide
SARS: Severe acute respiratory syndrome
SARS-CoV-2: Severe acute respiratory syndrome coronaviridae-2
SNP: Single nucleotide polymorphism
STAT: Signal transducer and activator of transcription
Th1: T-helper cell 1
Th2: T-helper cell 2
TGFβ: Transforming growth factor beta
TNF-α: Tumor necrosis factor-alpha

## References

[1] Ludvigsson JF (2020) Systematic review of COVID-19 in children shows milder cases and a better prognosis than adults. Acta Paediatr Int J Paediatr:1088–1095. 10.1111/apa.15270 10.1111/apa.15270 PMC7228328 32202343

[2] WHO (2020). Coronavirus disease. World Health Organ.

[3] Laboratories C, Surveillance NS (2020). Outpatient and Emergency Department Visits Severe Disease Summary of Laboratory Testing Results Reported to CDC *.

[4] Jones VG et al (2020) Pre-publication Release COVID-19 and Kawasaki Disease: Novel Virus and Novel Case Pre-publication Release Pre-publication Release. Hosp Pediatr Cit. 10.1542/hpeds.2020-0123 10.1542/hpeds.2020-0123 32265235

[5] Barron KS (2000). Kawasaki disease. Inflammatory Diseases of Blood Vessels.

[6] Burgner D, Harnden A (2005) Kawasaki disease: What is the epidemiology telling us about the etiology? Int J Infect Dis. 10.1016/j.ijid.2005.03.002 10.1016/j.ijid.2005.03.002 PMC7110839 15936970

[7] Sharma D, Singh S (2015). Kawasaki disease – A common childhood vasculitis. Indian J Rheumatol.

[8] Rowley AH (2000). IgA Plasma Cell Infiltration of Proximal Respiratory Tract, Pancreas, Kidney, and Coronary Artery in Acute Kawasaki Disease. J Infect Dis.

[9] Rowley AH, Shulman ST, Spike BT, Mask CA, Baker SC (2001). Oligoclonal IgA Response in the Vascular Wall in Acute Kawasaki Disease. J Immunol.

[10] Rowley AH (2005). Cytoplasmic Inclusion Bodies Are Detected by Synthetic Antibody in Ciliated Bronchial Epithelium during Acute Kawasaki Disease. J Infect Dis.

[11] Chang LY (2014). Viral infections associated with Kawasaki disease. J Formos Med Assoc.

[12] Kim GB (2014). Evaluation of the temporal association between Kawasaki disease and viral infections in South Korea. Korean Circ J.

[13] Nigro G (1994). Active or recent parvovirus B19 infection in children with Kawasaki disease. Lancet.

[14] Weng KP (2018). Enterovirus Infection and Subsequent Risk of Kawasaki Disease: A Population-based Cohort Study. Pediatr Infect Dis J.

[15] Maggio MC (2019). Kawasaki disease triggered by parvovirus infection: An atypical case report of two siblings. J Med Case Rep.

[16] Schnaar DA, Bell DM (1982). Kawasaki Syndrome in Two Cousins With Parainfluenza Virus Infection. Am J Dis Child.

[17] Lee D-H, Huang H-P (2004). Kawasaki disease associated with chickenpox: report of two sibling cases. Acta Paediatr Taiwan.

[18] Bajolle F (2014). Markers of a recent bocavirus infection in children with Kawasaki disease: ‘A year prospective study’. Pathol Biol.

[19] Shike H (2005). Adenovirus, Adeno-associated virus and kawasaki disease. Pediatr Infect Dis J.

[20] Embil JA, McFarlane ES, Murphy DM, Krause VW, Stewart HB (1985). Adenovirus type 2 isolated from a patient with fatal Kawasaki disease. Can Med Assoc J.

[21] Shirato K (2014). Possible involvement of infection with human coronavirus 229E, but not NL63, in Kawasaki disease. J Med Virol.

[22] Giray T (2016). Four cases with kawasaki disease and viral infection: Aetiology or association?. Infez Med.

[23] Jones VG et al (2020) COVID-19 and Kawasaki Disease: Novel Virus and Novel Case. Hosp Pediatr. 10.1542/hpeds.2020-0123 10.1542/hpeds.2020-0123 32265235

[24] Rowley AH (2004). Detection of Antigen in Bronchial Epithelium and Macrophages in Acute Kawasaki Disease by Use of Synthetic Antibody. J Infect Dis.

[25] McIntosh K (2005). Coronaviruses in the Limelight. J Infect Dis.

[26] Chesser H, Chambliss JM, Zwemer E (2017). Case Report Acute Hemorrhagic Edema of Infancy after Coronavirus Infection with Recurrent Rash.

[27] Hwang HH, Lim IS, Choi BS, Yi DY (2018) Analysis of seasonal tendencies in pediatric Henoch–Schönlein purpura and comparison with outbreak of infectious diseases. Medicine (United States) 97 10.1097/MD.0000000000012217 PMC6133644 30200139

[28] Gedalia A (2004). Henoch-Schönlein Purpura. Curr Rheumatol Rep.

[29] Ding Y (2003). The clinical pathology of severe acute respiratory syndrome (SARS): a report from China. J Pathol.

[30] Dhillon R et al (1996) Endothelial dysfunction late after Kawasaki disease. Circulation. 10.1161/01.CIR.94.9.2103 10.1161/01.cir.94.9.2103 8901658

[31] Ishikawa T, Seki K (2018). The association between oxidative stress and endothelial dysfunction in early childhood patients with Kawasaki disease. BMC Cardiovasc Disord.

[32] Poor HD et al (2020) COVID-19 Critical Illness Pathophysiology Driven by Diffuse Pulmonary Thrombi and Pulmonary Endothelial Dysfunction Responsive to Thrombolysis. medRxiv. 10.1101/2020.04.17.20057125 10.1002/ctm2.44 PMC7288983 32508062

[33] Rowley AH, Shulman ST (2018). The epidemiology and pathogenesis of Kawasaki Disease. Front Pediatr.

[34] Su S (2016). Epidemiology, Genetic Recombination, and Pathogenesis of Coronaviruses. Trends Microbiol.

[35] Rothan HA, Byrareddy SN (2020) The epidemiology and pathogenesis of coronavirus disease (COVID-19) outbreak. J Autoimmun. 10.1016/j.jaut.2020.102433 10.1016/j.jaut.2020.102433 PMC7127067 32113704

[36] Pan Y, Lu H (2017). Angiotensin-converting enzyme insertion/deletion polymorphism and susceptibility to Kawasaki disease: A meta-analysis. Afr Health Sci.

[37] Delanghe JR, Speeckaert MM, De Buyzere ML (2020) COVID-19 infections are also affected by human ACE1 D/I polymorphism. Clin Chem Lab Med:1–2. 10.1515/cclm-2020-0425 10.1515/cclm-2020-0425 32286246

[38] Nguyen A et al (2020) Human leukocyte antigen susceptibility map for SARS-CoV-2. J Virol. 10.1128/jvi.00510-20 10.1128/JVI.00510-20 PMC7307149 32303592

[39] Oh JH (2008). Polymorphisms of human leukocyte antigen genes in Korean children with Kawasaki disease. Pediatr Cardiol.

[40] Kumrah R, Vignesh P, Rawat A, Singh S (2020) Immunogenetics of Kawasaki disease. Clin Rev Allergy Immunol. 10.1007/s12016-020-08783-9 10.1007/s12016-020-08783-9 32200494

[41] Chand Dakal T et al (2020) Mechanistic basis of co-stimulatory CD40-CD40L ligation mediated regulation of immune responses in cancer and autoimmune disorders. Immunobiology. 10.1016/j.imbio.2019.151899 10.1016/j.imbio.2019.151899 31899051

[42] Gong J et al (2020) Correlation Analysis Between Disease Severity and Inflammation-related Parameters in Patients with COVID-19 Pneumonia. medRxiv:2020.02.25.20025643. 10.1101/2020.02.25.20025643 10.1186/s12879-020-05681-5 PMC7750784 33349241

[43] Teraura H (2017). The serum concentration of soluble interleukin-2 receptor in patients with Kawasaki disease. Ann Clin Biochem.

[44] Del Principe D (2017). Pathogenetic determinants in Kawasaki disease: the haematological point of view. J Cell Mol Med.

[45] Xue LJ et al (2017) Effect and Safety of TNF Inhibitors in Immunoglobulin-Resistant Kawasaki Disease: a Meta-analysis. Clin Rev Allergy Immunol. 10.1007/s12016-016-8581-4 10.1007/s12016-016-8581-4 27550227

[46] Chen W (2020). A potential treatment of COVID-19 with TGF-β blockade. Int J Biol Sci.

[47] Tanaka T, Narazaki M, Kishimoto T (2014) IL-6 in Inflammation, Immunity, and Disease. 6:1–16 10.1101/cshperspect.a016295 PMC4176007 25190079

[48] Kuo HC et al (2010) Serum albumin level predicts initial intravenous immunoglobulin treatment failure in Kawasaki disease. Acta Paediatr Int J Paediatr. 10.1111/j.1651-2227.2010.01875.x 10.1111/j.1651-2227.2010.01875.x 20491705

[49] Ueno Y (1989). The acute phase nature of interleukin 6: studies in Kawasaki disease and other febrile illnesses. Clin Exp Immunol.

[50] Lin CY, Lin CC, Hwang B, Chiang BN (1993). Cytokines predict coronary aneurysm formation in Kawasaki disease patients. Eur J Pediatr.

[51] Gauldie J, Richards C, Harnish D, Lansdorp P, Baumann H (1987). Interferon beta 2/B-cell stimulatory factor type 2 shares identity with monocyte-derived hepatocyte-stimulating factor and regulates the major acute phase protein response in liver cells. Proc Natl Acad Sci U S A.

[52] Wu Y et al (2019) Interleukin-6 is prone to be a candidate biomarker for predicting incomplete and IVIG nonresponsive Kawasaki disease rather than coronary artery aneurysm. Clin Exp Med. 10.1007/s10238-018-00544-5 10.1007/s10238-018-00544-5 30617865

[53] Nandi A, Pal P, Basu S (2019). A comparison of serum IL6 and CRP levels with respect to coronary changes and treatment response in Kawasaki disease patients: a prospective study. Rheumatol Int.

[54] Fan C, Li K, Ding Y, Lu WL, Wang J (2020) ACE2 Expression in Kidney and Testis May Cause Kidney and Testis Damage After 2019-nCoV Infection. medRxiv:2020.02.12.20022418. 10.1101/2020.02.12.20022418

[55] Booz GW, Day JNE, Baker KM (2002). Interplay between the cardiac renin angiotensin system and JAK-STAT signaling: Role in cardiac hypertrophy, ischemia/reperfusion dysfunction, and heart failure. J Mol Cell Cardiol.

[56] Qi YF et al (2016) Angiotensin-converting enzyme 2 inhibits high-mobility group box 1 and attenuates cardiac dysfunction post-myocardial ischemia. J Mol Med. 10.1007/s00109-015-1356-1 10.1007/s00109-015-1356-1 PMC4842346 26498282

[57] Conti P et al (2020) Induction of pro-inflammatory cytokines (IL-1 and IL-6) and lung inflammation by COVID-19: anti-inflammatory strategies. J Biol Regul Homeost Agents. 10.23812/CONTI-E 10.23812/CONTI-E 32171193

[58] Rizzo P (2020). COVID-19 in the heart and the lungs: could we “Notch” the inflammatory storm?. Basic Res Cardiol.

[59] AbdelMassih, A. et al. Is it infection or rather vascular inflammation ? Game-changer insights and recommendations from patterns of multi-organ involvement and affected subgroups in COVID-19, Cardiovascular Endocrinology & Metabolism: June 11, 2020 - Volume Publish Ahead of Print - Issue - 10.1097/XCE.0000000000000211 10.1097/XCE.0000000000000211 PMC7410022 32803145

[60] AbdelMassih, A. et al. Obese communities among the best predictors of COVID-19-related deaths. Cardiovascular Endocrinology & Metabolism: June 11, 2020 - Volume Publish Ahead of Print - Issue - 10.1097/XCE.0000000000000218 10.1097/XCE.0000000000000218 PMC7314342 32803143

[61] AbdelMassih, A. et al. Possible molecular and paracrine involvement underlying the pathogenesis of COVID-19 cardiovascular complications, Cardiovascular Endocrinology & Metabolism: April 20, 2020 - Volume Publish Ahead of Print - Issue - 10.1097/XCE.0000000000000207 10.1097/XCE.0000000000000207 PMC7410028 32803146

[62] Luo P et al (2020) Tocilizumab treatment in COVID-19: A single center experience. J Med Virol. 10.1002/jmv.25801 10.1002/jmv.25801 PMC7262125 32253759

[63] Nozawa T, Imagawa T, Ito S (2017) Coronary-artery aneurysm in tocilizumab-treated children with Kawasaki’s disease: To the editor. N Engl J Med. 10.1056/NEJMc1709609 10.1056/NEJMc1709609 29117496

